# Lycopene Prevents Development of Steatohepatitis in Experimental Nonalcoholic Steatohepatitis Model Induced by High-Fat Diet

**DOI:** 10.4061/2010/262179

**Published:** 2010-10-03

**Authors:** Ibrahim Halil Bahcecioglu, Nalan Kuzu, Kerem Metin, Ibrahim Hanifi Ozercan, Bilal Ustündag, Kazim Sahin, Omer Kucuk

**Affiliations:** ^1^Division of Gastroenterology, School of Medicine, Firat University, 23200 Elazig, Turkey; ^2^Department of Biochemistry, School of Medicine, Firat University, 23200 Elazig, Turkey; ^3^Department of Pathology, School of Medicine, Firat University, 23200 Elazig, Turkey; ^4^Department of Animal Nutrition, Faculty of Veterinary Medicine, Firat University, 23200 Elazig, Turkey; ^5^Winship Cancer Institute, Emory University, Atlanta, GA 30322, USA

## Abstract

We investigated the preventive effect of lycopene on nonalcoholic steatohepatitis-induced by high-fat diet in rats. Forty male Sprague-Dawley rats were divided into 4 groups. They were fed standard diet, high-fat diet (HFD), high-fat diet plus lycopene at a dose of 2 mg/kg body weight and the high-fat diet lycopene at a dose of 4 mg/kg BW for a period of 6 weeks. Inflammation, steatosis, *α*-smooth muscle actin (*α*-SMA), and cytochrome P450 2E1 (CYP 2E1) expression increased significantly in the rats fed HFD and decreased in the rats administered by lycopene. Significantly elevated levels of serum alanine aminotransferase (ALT), aspartate aminotransferase (AST), tumor necrosis factor (TNF *α*), and serum and liver malondialdehyde (MDA) were observed in rats fed the high-fat diet as compared to the control rats (*P* < .01). Supplementation with lycopene lowered serum MDA and tumor necrosis factor (TNF-*α*) levels and elevated liver GSH level (*P* < .001). Insulin resistance was higher in the rats fed HFD than in rats supplemented with lycopene. The data indicate that supplementation with lycopene can reduce high-fat diet-induced oxidative stress to the cells.

## 1. Introduction

 Nonalcoholic steatohepatitis (NASH) histologically resembles alcoholic steatohepatitis. It is characterized by diffuse fatty infiltration in the liver, and ballooning degeneration and inflammation in the hepatocytes. Histopathological signs may be accompanied by Mallory's hyaline and perisinusoidal fibrosis [1–4]. There may be progression to cirrhosis [5, 6]. Experimental model of NASH has enabled the understanding of its pathogenesis. These were induced in genetically obese ob/ob mice, lipoatrophic mice, and experimental animals fed on methionine-deficient diet (MCD). All models appear to support multiple hit hypotheses [7]. In the experimental models, steatosis is rapidly formed in nonalcoholic fatty liver disease (NAFLD) models induced in ob/ob mice (obese, and having dyslipidemia and insulin resistant diabetes) and lipoatrophic rats, and with methionine- and choline-deficient diet. Its progression to steatohepatitis and fibrosis is highly variable [8]. In the ob/ob mice model, there is only steatosis at the onset. Liver injury emerges in case of leptin administration and/or exposure to secondary harmful effects. As ob/ob and lipoatrophic mice have leptin deficiency, cirrhosis generally does not develop in them. Methionine- and choline-deficient diet leads to steatohepatitis by reducing antioxidant defense systems and increasing oxidative stress. There is a nutritional deficiency in methionine- and choline-deficient diet. However, there is no such defect in human NAFLD. Human NAFLD is mostly associated with obesity and diabetes. Therefore, the models cited previously are not ideal experimental models.

 High-fat diet (HFD) was seen to produce signs resembling NASH in humans. In a standard diet, 35% of the energy is procured from fat, 18% from protein, and 47% from carbohydrate, while in HFD, 71% of the energy is obtained from fat, 11% from carbohydrate, and 18% from protein [9]. Peripheral insulin resistance, increased fatty acid beta-oxidation, increase in oxidative stress in the liver, and fatty degeneration in the liver are known characteristics of NASH [3–6]. In a study with HFD, an increase in hepatic TNF *α*, TNF *α* mRNA, oxidative stress, and cytochrome P450 mRNA levels were found together with histological signs of steatohepatitis at the end of 3 weeks [9].

 Lycopene is a carotenoid that is found abundantly in tomatoes and has strong antioxidant characteristics [10, 11]. Previous studies showed that intake of tomatoes and tomato products strengthened the antioxidant system, and inhibited lipid peroxidation in humans [12]. Besides, it was demonstrated that oral lycopene administration for 2 weeks inhibited lipid peroxidation in the liver tissues of rats [13]. The objective of present study was to investigate the protective effect of lycopene on the development of steatohepatitis in NASH model induced by HFD.

## 2. Material and Method

### 2.1. Animals and Groups

Forty male Sprague-Dawley rats, which were purchased from Firat University (Elazig, Turkey), were maintained under controlled environmental conditions (temperature 23 ± 2°C, relative humidity 55%± 10%, 12/12 h light-dark cycle) and given food and water ad libitum. All rats were acclimated for 1 week prior to the experiment. Four groups of 10 rats each were treated with their respective group diet for 6 weeks. The control group was fed a standard diet. The HFD group was fed a high-fat liquid diet (HFD; using AIN76A diet as the base by Dyets, Inc., Bethlehem, PA) with 71% of energy derived from fat, 11% from carbohydrates, and 18% from protein. The other two experimental groups were fed the same high-fat diet with lycopene (DSM, Istanbul, Turkey) at a dose of 2 mg/kg body weight (HFD + 2 mg/kg lycopene group) and at a dose of 4 mg/kg body weight (HFD + 4 mg/kg lycopene group). Lycopene was administered by gastric gavage mixed with olive oil and given 3 days per week. The diets were isocaloric and isonitrogenic. This study and all procedures were approved by the Animal Care and Use Committee of Firat University, Turkey. The study lasted for 6 weeks, and the rats were sacrificed by decapitation. Liver samples were collected from the animals for biochemical and histopathological examination.

### 2.2. Plasma and Tissue MDA Measurements

Plasma MDA levels were measured in accordance with thiobarbituric acid method, modified by Yagi [14]. Results were presented as nmol/mL. Liver tissue MDA levels were analyzed by Okhawa method. Results were expressed as nmol/g tissue [15].

### 2.3. Glutathione Level

Determination of GSH was carried out as described by Sedlak and Lindsay [16]. Results were shown as *μ*mol/mg tissue.

### 2.4. TNF-*α* Levels

TNF *α* was determined by using a commercial kit (Biosoruce immunoassay kit, Biosource international, Inc., CA., USA) in a spectrophotometer with ELISA (EL_x_-800, Bio-Tek Instruments Inc., City, VT).

### 2.5. Biochemical Parameters

Blood samples collected from the rats were centrifuged to obtain plasma, which was kept at −20°C until analysis. Insulin was analyzed using ELISA method, while fasting blood glucose, alanine aminotransferase (ALT), aspartate aminotransferase (AST), alkaline phosphatase (ALP), gamma glutamyl peptidase (*γ*-GT), triglyceride, and cholesterol levels were studied in Olympus AU 600 autoanalyzer. Insulin resistance was calculated according to the formula [17]. HOMA-IR: [serum glucose level (mg/dL)/18.1xinsulin level (*μ*IU/mL)]/22.5. A high HOMA-IR score indicates high insulin resistance.

### 2.6. Histopathological Examination

Liver tissue samples were stored in 10% formalin solution. Paraffin blocks were prepared. Cross-sections obtained from the blocks were stained with hematoxylin eosin and Masson's trichrome. Histopathological examination was carried out by a specialist pathologist. Steatohepatitis was diagnosed according to Brunt's histopathological criteria [4]. Histopathological signs of steatosis, inflammation, and fibrosis were scored semiquantitatively and were modified from Brunt's criteria [18, 19]. Percentage of steatotic cells was determined. They were graded as + up to 25%, ++ between 26% and 50%, +++ between 51% and 75%, and ++++ >76%. Inflammatory cells were counted in randomly chosen 10 areas under ×400 magnification; the total number was divided by 10 to determine the mean number of inflammatory cells per mm^2^. Necrosis was counted in randomly chosen 10 areas under ×400 magnification, and the total was divided by ten to determine the mean necrotic foci per mm^2^. Ballooning degeneration and Mallory's body were evaluated on the basis of the presence/absence. Cross-sections taken from paraffin blocks were stained with Mason's Trichrome, and examined under ×40, ×100, ×200, and ×400 magnification. Fibrosis was staged between 0 and 4. Pericellular fibrosis in Zone 3 stage 1; perivenular and pericellular fibrosis in zone 2 and 3, irrespective of the presence of portal and/or periportal fibrosis stage 2; bridging fibrosis stage 3; cirrhosis stage 4.

### 2.7. Immunohistochemical Examination

Immunohistochemical *α*-smooth muscle actin (*α*-SMA) staining was conducted in liver tissue to show hepatic stellate cell (HSC) activation (Actin smooth muscle neomakers, catalogue no: RU-910-R7). Presence of HSC reactive *α*-sMA in the liver was scored semiquantitatively [20]. Grade 0: No or very rare staining, grade 1: staining of <30% of stellate cells in sinusoidal liver cells, grade 2: staining between 31% and 60% of cells, grade 3: staining between 61% and 90% of cells, and grade 4: diffuse staining in more than 90% of sinusoidal liver cells.

Immunohistochemical staining was applied to liver tissue to show CYP2E1 expression (CYP2E1 Chemicon catalogue no: AB1252). Zonal staining score was determined as perivenular staining: 1, midzonal staining: 2, and panlobular staining: 3. Severity of staining was evaluated as no staining: 0, mild staining: 1, moderate staining: 2, and severe staining: 3 [21].

### 2.8. Statistical Evaluation

Data presented in the study were shown as mean ± standard deviation, unless indicated otherwise. Paired *T* test was used in the evaluation of data, and Mann Whitney *U* test in double evaluations. Additionally, Pearson Spearman correlation tests were employed for some parameters. SPSS 11.0 package software was used in statistical evaluations.

## 3. Results

Body and liver weights are presented in Table 1. The increase in mean weight was significant in the treatment groups (HFD) in comparison to the control group (*P* < .05 for each). There was no significant difference in liver weight between the groups fed on the experimental diets for 6 weeks (*P* > .05, Table 1). 

### 3.1. Biochemical Parameters

The serum activities of ALT, AST, ALP, and levels of triglyceride and cholesterol in the rats fed with HFD group were significantly higher than those in the control and the lycopene groups (*P* < .05). However, the increase in the activity of these enzymes was markedly suppressed by supplementing lycopene (*P* < .05, Table 2) to the level of the normal control. However, the supplementation level of lycopene did not result in dose-dependency. ALT and triglyceride levels decreased when compared to rats fed HFD (*P* < .05 for each), while there was no difference in AST, ALP, and cholesterol levels (*P* > .05). ALT, AST, ALP, cholesterol, and triglyceride levels did not change between rats fed HFD and supplemented with different dose of lycopene (*P* > .05).

### 3.2. Oxidative Stress Measurement

Table 2 presents the significant increase in MDA level in serum and liver as a result of lipid peroxidation in the HFD rats (*P* < .001). However, lycopene supplementation significantly inhibited this lipid peroxidation when compared to the HFD group (*P* < .01) and returned it to the level of the normal control group. The MDA levels between the lycopene-supplemented groups were not significantly different (*P* < .001 for each, Table 3). There was no significant difference in the liver total glutathione concentration between control and HFD groups. However, lycopene supplementation tended to increase the concentration of total glutathione (*P* < .001, Table 3).

### 3.3. Insulin Resistance

Insulin resistance increased in rats fed HFD, compared to the control rats (*P* < .05), decreased in rats administered lycopene at 2 or 4 mg/kg level, but the difference was not statistically significant.

### 3.4. Serum TNF-*α* Level

The TNF-*α* level in the HFD group was significantly higher than that in the control (*P* < .05, Table 2). However, the TNF-*α* level in the lycopene-supplemented groups was significantly lower than the level in the HFD group (*P* < .05, Table 2) and similar to the level of the control. However, the supplementation level of lycopene did not significantly affect the TNF-*α* level.

### 3.5. Histopathological Examinations

**Figure 1:**
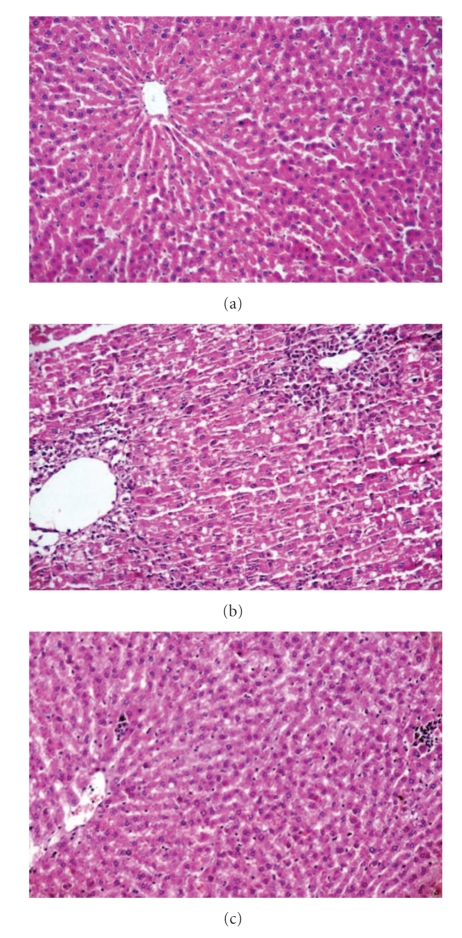
Histopathological findings in groups. (a) Normal liver histology from liver section rat fed on standart diet (H&E × 200). (b) Macro- and microvesicular steatosis and ballooning degeneration around the central vein from liver section rat fed on HFD (Hematoxylen-Eosin (H&E × 200). (c) The marked decreased steatosis and inflammation from liver section rat fed on HFD + lycopene (H&E × 200).

Histopathological findings are presented in Table 4 and Figure 1. Steatosis, inflammation, ballooning degeneration, and necrosis significantly increased rats fed HFD, compared to control. The difference was in the increase in fibrosis between control and HFD groups were not significant. Mallory's hyaline was found only in rats fed HFD (20%), but was not found in other groups. Steatosis and inflammation in rats supplemented with lycopene decreased compared to HFD rats (*P* < .05). The difference between HFD and HFD/lycopene (2 mg/kg) was not significant for ballooning degeneration, fibrosis, and necrosis (*P* > .05), nor there was any significant difference between lycopene doses.

### 3.6. Immunohistochemical Analysis

**Figure 2:**
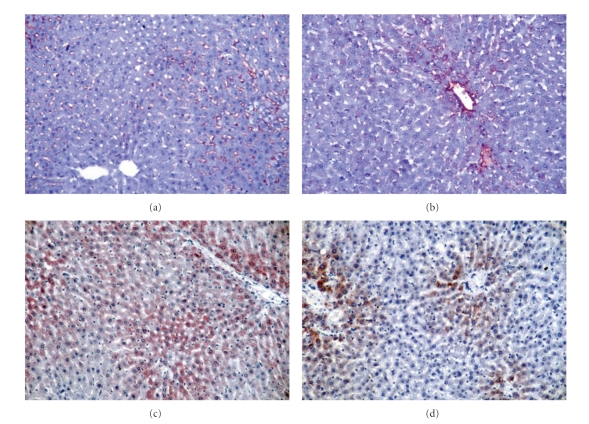
Immunohistochemical analysis. *α* SMA expression was increased liver sections rat fed on HFD (a), and decreased rat fed on HFD + lycopene (b) (original magnification ×200). CYP 2E1 expression was shown in localization of panlobuler rat fed on HFD (c), and perivenular by rat fed on HFD + lycopene (d) (original magnification ×200).

Immunohistochemical analysis is presented in Figure 2. Expression of *α*-sMA and CYP2E1 increased in rats fed HFD, compared to control rats (*P* < .001). CYP2E1 and *α*-sMA expression decreased in rats fed HFD and lycopene at 2 mg/kg, in comparison to rats fed HFD (*P* < .01); both *α*-sMA and CYP2E1 expression significantly decreased in rats fed HFD and lycopene at 4 mg/kg, when compared to rats HFD (*P* < .01). There was no significant difference between lycopene doses in terms of *α*-sMA and CYP2E1 expression (*P* > .05). There was a positive correlation between CYP2E1 expression, and plasma and liver tissue MDA increase (*r* = 0.704; *P* < .001, *r* = 0.559; *P* < .001, resp.). There was no significant correlation in terms of liver glutathione levels.

## 4. Discussion

Lycopene is the predominant carotenoid found in tomatoes and tomato products [22, 23]. It has antioxidant and radical oxygen scavenging effects, through which it protects tissues and cells against harmful effects of free radicals [24, 25]. Consumption of large amounts of tomatoes was shown to improve the antioxidant status and inhibit lipid peroxidation [26]. The major organ where lycopene accumulates after single dose ^14^C-lycopene administration is the liver [27]. Its level in liver tissues of rats orally fed on lycopene was found to remain unchanged between 0.5 g/kg and 5 g/kg, but directly increased from 0 to 5 g/kg. It levels off in concentrations dissolved in 10% water between 0.05 g/kg and 0.5 g/kg [28]. In the present study, we found oral lycopene administration to have a preventive effect on the development of steatohepatitis in rats in which experimental NASH was induced. We established that steatosis and inflammation were significantly lower in lycopene-administered rats, compared to the rats fed on an HFD alone. There was not any difference between different doses of lycopene. This is the first study examining the preventive role of lycopene in experimental NASH model induced by HFD. Oxidative stress plays a major role in the pathogenesis of NASH [2, 8, 29]. Lipid peroxidation occurs through a mechanism associated with free radicals. This process leads to oxidative destruction of polyunsaturated fatty acids in cellular membranes [30]. Lycopene administration brought about a decrease in lipid peroxidation and an increase in liver glutathione levels. Among carotenoids, lycopene has the highest antioxidant capacity [31]. As a cellular antioxidant, glutathione conjugates various electrophilic byproducts that are able to initiate lipid peroxidation, and has the ability to directly scavenge free radicals [32]. While preventing oxidative stress by its free radical scavenging characteristic, glutathione strengthens antioxidant defense systems. 

High-fat diet intake is associated with increased oxidative stress [9] and induction of the cytochrome P4502E1 (CYP2E1) enzyme [29, 33]. CYP2E1 has enhanced NADPH oxidase activity resulting in increased production of the reactive oxygen species superoxide and hydrogen peroxide by redox cycling of endogenous and exogenous substrates [33, 34]. The absence of antioxidants has been shown to further promote high-fat diet-induced oxidative stress, which could lead to an inflammatory state. In the present study, we observed a decreased CYP2E1 protein with increasing lycopene dose in the high-fat diet rats. This decrease was significantly greater when lycopene was supplemented at the high dose in rats compared with the rats that were supplemented with the standard diet. Although the mechanisms for these results are currently unknown, our observation suggests that lycopene supplementation may alleviate the harmful effects of excessive high-fat diet intake. Lycopene has been shown to act as an antioxidant depending on the dose at which it is supplemented [23, 24]. Similarly, Louisa et al. [35] reported that CYP2E1 probe enzyme (p-nitrophenol hydroxylase) was significantly reduced by repeated administration of 100 mg/kgBW/day lycopene. In a study conducted with ethanol, ethanol caused an increase in hydrogen peroxide and overexpression of CYP2E1, and this increase was reduced by lycopene. Besides, glutathione levels decreased, which was parallel to the increase in hydrogen peroxide radicals [36]. In our study, CYP2E1 expression in rats with induced NASH increased, and this increase was inhibited by both doses of lycopene, as was the case in the alcoholic model. 

As HFD was continued for 6 weeks in the present study, fibrosis development in this period was minimal. Therefore, evaluation in terms of fibrosis was not adequate. Activation of HSC, which has the key role in the development of fibrosis, was examined by showing sMA expression through the immunohistochemical method. Lycopene was demonstrated to inhibit HSCs in experimental liver fibrosis model, and to suppress fibrogenesis [37]. It was seen in our study that lycopene administration inhibited *α*-sMA expression, as in liver fibrosis model.

Cytotoxic products of the lipid peroxidation can impair cellular functions, nucleotide and protein synthesis, and can have a part in liver fibrogenesis by modulating collagen genes in HSCs [38]. Most probably, lycopene suppresses HSC activation by inhibiting lipid peroxidation via its special free radical scavenging character. The primary metabolic abnormality involved in the progression from fatty liver to NASH is not known for sure. Insulin resistance plays a part in this progression [2]. Peripheral insulin resistance is among the well-known characteristics of NASH. NASH impairs the inhibiting effect of insulin on liver glucose output and its other metabolic effects. It leads to the activation of inhibitor kappa kinase beta (IKK-*β*), and thereby causes peripheral insulin resistance [39]. 

Many studies reported a central role of TNF-*α* and other proinflammatory cytokines in the development of obesity-associated insulin resistance and fatty liver [40, 41]. Schattenberg et al. [33] indicated that the presence of steatohepatitis result in the downregulation of insulin signaling, potentially contributing to the insulin resistance associated with nonalcoholic fatty liver disease. High-fat diet intake also leads to the activation of several proinflammatory cytokines, including TNF-a, and increased hepatic infiltration by inflammatory cells [9, 33]. TNF-*α* is an important cytokine in the development of many forms of liver injury, including steatohepatitis [40]. The interaction between cytokines, and oxidative stress and lipid peroxidation plays a key role in the development of steatohepatitis [41]. TNF *α*, a significant mediator in insulin resistance, affects the activity of tyrosine kinase in insulin receptors [42]. In the present study, we have shown that TNF-*α* level was reduced by the supplemental lycopene, indicating that inflammation was prevented. The groups that were administered lycopene also displayed a decrease in insulin resistance, although the decrease was not statistically significant. Similar to our results, it is reported that the adipose tissue secretes inflammatory cytokines such as TNF-*α* by inducing insulin resistance and upregulating the expression of other inflammatory mediators [43]. Additionally, Herzog et al. [44] reported that lycopene supplementation reduces transcript levels of proinflammatory cytokines with downregulation of IL-6 expression. 

In conclusion, lycopene has a preventive effect on the development of steatohepatitis in nonalcoholic steatohepatitis induced experimentally by HFD. This effect can be primarily explained by its antioxidant characteristic. There is no difference between the oral administration of 2 mg/kg and 4 mg/kg lycopene.

## Figures and Tables

**Table 1:** The effects of lycopene supplementation on body weight and liver weight in groups.

Weight	Control group (*n* = 10)	HFD (*n* =10)	HFD + 2 mg/kg lycopene (*n* = 9)	HFD + 4 mg/kg lycopene (*n* = 10)
Baseline BW	299,80 ± 40,29	297,90 ± 26,34	300,70 ± 30,22	298,20 ± 27,89
Final BW	328,30 ± 37.5	360,70 ± 20,58**	364,30 ± 42,10	375,70 ± 34,24*
Liver weight (gr)	9,41 ± 1,10	10,05 ± 1,38	9,75 ± 1,40	10,15 ± 1,36

BW: body weight, *P < .05: compared with HFD group; **P < .05: compared with control.

**Table 2:** The effects of lycopene on serum parameters in groups.

Parameter	Control group (*n* = 10)	HFD (*n* = 10)	HFD + 2 mg/kg lycopene (*n* = 9)	HFD + 4 mg/kg lycopene (*n* = 10)
Insulin (*μ*IU/mL )	0,129 ± 0,069	0,132 ± 0,028	0,116 ± 0,340	0,102 ± 0,0230
HOMA-R	0.032 ± 0,02	0,043 ± 0,02	0,036 ± 0.01	0,034 ± 0.06
Glucose (mg/dL)	127,33 ± 12,30	139,11 ± 9,25	130,22 ± 8,89*	139,55 ± 14,53
Triglyceride (mg/dL)	67,2±14,2	189,5 ± 93,7^†^	157,3 ± 56,8	130,9 ± 33,3*
Cholesterol (mg/dL)	49,80 ± 5,76	71,7 ± 6,2	78,4 ± 20.2	71,0 ± 5,18
ALT (IU/L)	84,1 ± 7,3	96,2 ± 11,4^†^	85,8 ± 7,4*	81,2 ± 15,7*
AST (IU/L)	174,4 ± 14,5	247,3 ± 21,7^†^	231,7 ± 38,5	238,0 ± 20,16
ALP (IU/L)	213,3 ± 63,7	467,3 ± 87,9^†^	440,5 ± 108,0	414,5 ± 113,3
GGT (IU/L)	1,3 ± 0,4	1,8 ± 1,2	1.1 ± 0.60	1,7 ± 0.9^†^
TNF-*α* (pg/mL)	0,192 ± 0,50	0,615 ± 0,358^†^	0,226 ± 0,126*	0,254 ± 0,186*

*P < .05: compared with HFD group; ^†^ *P* < .05: compared with control.

**Table 3:** The effects of lycopene supplementation on MDA and glutathione levels in groups.

Parameter	Control group (*n* = 10)	HFD (*n* = 10)	HFD + 2 mg/kg lycopene (*n* = 9)	HFD + 4 mg/kg lycopene (*n* = 10)
Plasma MDA (nmol/mL)	2,06 ± 0.54	4,28 ± 0.94**^†^**	2,58 ± 0,54*	2,12 ± 0,51*
Liver MDA (nmol/gr)	20,72 ± 2.70	29,8 ± 3,91**^†^**	20,21 ± 4,39*	15,89 ± 4,65*
Liver glutathione (*μ*mol/mg)	9,07 ± 1,01	8,08 ± 1,19	22,62 ± 20,09*	20,96 ± 3,91*

*P < .001: compared with high-fat diet group; ^†^ *P* < .001: versus control group.

**Table 4:** The effects of lycopene supplementation on histopathological changes.

Parameters	Control group (*n* = 10)	HFD (*n* = 10)	HFD + 2 mg/kg lycopene (*n* = 9)	HFD + 4 mg/kg lycopene (*n* = 10)
Steatosis (0–4)	—	1,30 + 0,48^†^	0,6 ± 0,51*	0,70 ± 0,48*
İnflammation (cells/mm^2^)	2,11±1,07	12,45 ± 6,0^†^	4,50 ± 2,1**	4,95, ±3,81**
Necrosis (foci/mm^2^)	—	1,15 ± 0,87^†^	0,72 ± 0,24	0,57 ± 0,38
Fibrosis (0–4)	—	0,50 ± 0,52^†^	0,60 ± 0,51	0,20 ± 0,42
CYP 2E1 expression	1,0 ± 0,0	2,60 ± 0,51^†^	1,70 ± 0,48**	1,20 ± 0,42**
*α*-SMA expression	—	2,50 ± 0,52^†^	1,80 ± 0,62*	1,40, ±0,16**

*P < .05: versus HFD group, **P < .01: versus HFD group; versus HFD group, †*P* < .01: versus control group.
